# Biopolymeric Ni_3_S_4_/Ag_2_S/TiO_2_/Calcium Alginate Aerogel for the Decontamination of Pharmaceutical Drug and Microbial Pollutants from Wastewater

**DOI:** 10.3390/nano12203642

**Published:** 2022-10-17

**Authors:** Rajeev Kumar, Mohammad Oves, Mohammad Omaish Ansari, Md. Abu Taleb, Mohamed Abou El-Fetouh Baraka, Mansour A. Alghamdi, Naief Hamoud Al Makishah

**Affiliations:** 1 Department of Environmental Sciences, Faculty of Meteorology, Environment and Arid Land Agriculture, King Abdulaziz University, Jeddah 21589, Saudi Arabia; 2 Central of Excellence in Environmental Studies, King Abdulaziz University, Jeddah 21589, Saudi Arabia; 3 Central of Nanotechnology, King Abdulaziz University, Jeddah 21589, Saudi Arabia

**Keywords:** Ni_3_S_4_/Ag_2_S/TiO_2_/CA aerogel, drug adsorption, microbial decontamination, photocatalysis, mechanism

## Abstract

The ubiquitous presence of pharmaceutical drugs and microbes in the water is leading to the development of drug resistant microbes. Therefore, efficient materials that can remove or inactivate the drug and microbe contaminants are required. In this work, nickel sulfide/calcium alginate (Ni_3_S_4_/CA), silver sulfide/calcium alginate (Ag_2_S/CA), modified titanium dioxide/calcium alginate (TiO_2_/CA), and Ni_3_S_4_/Ag_2_S/TiO_2_/calcium alginate (Ni_3_S_4_/Ag_2_S/TiO_2_/CA) aerogels have been synthesized for the removal of the oxytetracycline (OTC) drug and microbial contaminants from real beverage industry wastewater. The results revealed that Ni_3_S_4_/Ag_2_S/TiO_2_/CA aerogel is highly efficient for OTC adsorption and inactivation of microbes compared to Ni_3_S_4_/CA, Ag_2_S/CA and TiO_2_/CA aerogels. The OTC adsorption depends greatly on the solution pH, and optimum OTC removal was observed at pH 6 in its zwitterionic (OTC^±^) form. The formation of H-bonding and n-π electron donor-acceptors is possible to a considerable extent due to the presence of the double bond benzene ring, oxygen and nitrogen, sulfur-containing functional groups on the OTC molecules, and the Ni_3_S_4_/Ag_2_S/TiO_2_/CA aerogel. Based on the statistical analysis, root-mean-square deviation (RMSD), chi square (χ^2^) values, and higher correlation coefficient (R^2^) values, the Redlich–Peterson isotherm model and Elovich kinetic model are most suited to modelling the OTC adsorption onto Ni_3_S_4_/Ag_2_S/TiO_2_/CA. The prepared aerogels’ excellent antimicrobial activity is observed in the dark and with solar light irradiation. The zone of inhibition analysis revealed that the antimicrobial activity of the aerogels is in the following order: Ni_3_S_4_/Ag_2_S/TiO_2_/CA > TiO_2_/CA > Ag_2_S/CA > Ni_3_S_4_/CA, respectively. Moreover, the antimicrobial results demonstrated that reactive oxygen species, electrons, and active radical species are responsible for growth inhibition and killing of the microbes. These results indicated that Ni_3_S_4_/Ag_2_S/TiO_2_/CA aerogel is highly efficient in decontaminating pollutants from wastewater.

## 1. Introduction

The complex nature of the wastewater released from industries and domestic activity is challenging traditional wastewater treatment methods. Municipal wastewater is now more complex and contains organic, inorganic, and microbial pollutants. In fact, the presence of pharmaceuticals and microbes is very common in municipal wastewater. The presence of pharmaceutical drugs and bacteria is not safe for human health because bacteria may develop resistance to the drugs [1]. The problem of pharmaceuticals and microbial pollution is growing as the consumption and release of these pollutants surges. In this context, nanomaterials derived from metal oxides, carbon, polymers, biopolymers, solid waste, etc. are being investigated for the decontamination of pharmaceuticals and microbial pollutants [2]. The removal of these pollutants using nanomaterials is mainly controlled by surface interaction or decomposition. However, synthesizing nanomaterials with high decontamination and reusable properties is still challenging.

Metal sulfides and their composite materials are widely investigated in energy, catalysis, and environmental remediation applications [3,4]. Previous studies reported that sulfides of copper, silver, molybdenum, zinc, nickel, selenium, etc., had been used to remove heavy metals and organics, and for disinfection [5]. The high efficacy of the metal sulfides for removal of contaminants is due to their structure, with many active sites and defects, S and metal edge sites, and basal plane sites [6]. In metal sulfides, metals and sulfur atoms are bonded chemically to make a stable structure. Therefore, the properties and structure of the metal sulfide can be tuned to develop an efficient material for water purification. Silver and nickel sulfides are well-known materials for environmental remediation applications. Kumar et al. [7] synthesized polyacrylamide grafted gum karaya/NiS/Ni_3_S_4_ bio-nanocomposite to remove rhodamine 6G, showing a very high adsorption capacity (1244.71 mg/g). Alongside the adsorption properties, the antimicrobial properties of pure or Ni_x_S_y_-based composites have been reported in the literature. Hosseini et al. [8] fabricated a NiS-SiO_2_ nanocatalyst and investigated its efficacy as a photocatalyst and antimicrobial agent. The synthesized material was highly stable in UV light and performed better than the parent materials. Highly stable hollow/solid Ag_2_S/Ag heterodimers were synthesized by ion exchange and photo-assisted reduction. Ag_2_S/Ag heterodimers showed excellent deactivation properties of *Escherichia coli* K-12 [9]. Besides the antimicrobial application, Ag_2_S@Ag nanoparticles have been investigated for superior dye adsorption performance by Zhang et al. (2016) [10]. Gupta et al. [11] used the Ag_2_S-chitosan nanohybrid for Clindamycin antibiotic sorption. The electrostatic interaction between the drug and nanohybrid was a major driving force in the sorption process. The adsorption of the organic pollutants onto metal sulfide is controlled by ionic bond formation, hydrogen bonding, and electron donor-acceptor mechanisms. At the same time, the microbes’ deactivation occurs by cell destruction caused by eluted metal ions’ cytotoxicity and active radicals produced by the material. Although the leaching of the metal ions from the materials showed excellent microbial deactivation, the nanocatalyst’s stability is compromised and reusability of the material is limited [9]. Therefore, the development of sustainable and economical metal sulfide-based materials with high stability, easy separation from the aqueous solution, and excellent adsorption and microbial properties for wastewater purification is needed.

Developing new mixed metal sulfides or combinations with metal oxides or polymers having good adsorption and antibacterial effects could have advantages over the single component materials. Therefore, synthesizing the mixed silver and nickel sulfide (Ni_3_S_4_/Ag_2_S) on a TiO_2_ surface could be a new approach for the fabrication of a new material (Ni_3_S_4_/Ag_2_S/TiO_2_) for the pharmaceutical drug and microbial decontamination for wastewater. However, separating the powdered Ni_3_S_4_/Ag_2_S/TiO_2_ nanocomposite might be challenging. In this context, synthesizing an aerogel could be a better approach to develop porous materials that can easily be separated from the aqueous solution and enhance the water purification capacity of the immobilized material. Polysaccharides like alginate have special advantages because of their structural and physicochemical properties, biocompatibility, environmentally friendliness, good adsorptive and antimicrobial capacity [12]. The immobilization of Ni_3_S_4_/Ag_2_S/TiO_2_ nanocomposite within the alginate matrix could show higher adsorption and microbial decontamination properties, and its aerogel can be easily isolated from the wastewater after use. Herein, Ni_3_S_4_/Ag_2_S/TiO_2_/calcium alginate (Ni_3_S_4_/Ag_2_S/TiO_2_/CA) aerogel has been synthesized to remove the oxytetracycline (OTC) drug and promote microbial decontamination of waste water from the beverage industry. A detailed mechanism has been proposed to understand the adsorption and microbial decontamination behaviour of the Ni_3_S_4_/Ag_2_S/TiO_2_/CA.

## 2. Materials and Method

### 2.1. Materials

Sodium alginate was received from Techno Pharma (Delhi, India). TiCl_4_, Na_2_S, NiCl_2_, silver acetate, and CaCl_2_ were obtained from Sigma-Aldrich (Saint-Louis, MO, USA), and BDH Chemicals Ltd. (Mumbai, India). TiO_2_ (P25) was obtained from Alfa Aesar (Haverhill, MA, USA).

### 2.2. Synthesis

#### 2.2.1. Modification of TiO_2_

Initially, 5 g TiO_2_ (P25) was mixed with 70 mL 10 M NaOH and heated at 120 °C in a hydrothermal reactor for 24 h. Obtained sodium titanate was washed with deionized water to remove excessive Na^+^ ions and dried in an oven dryer for 24 h at 105 °C. After that, sodium titanate was calcined at 400 °C for 3 h. The obtained material was washed with water and ethanol and dried at 105 °C.

#### 2.2.2. Synthesis of Ni_3_S_4_/Ag_2_S/TiO_2_ Nanocomposite

A thermal method was used for the synthesis of Ni_3_S_4_/Ag_2_S/TiO_2_. Initially, 1.51 g Na-TiO_2_ (sodium titanate), 0.62 g NiCl_2_, and 0.27 g silver acetate were mixed in an agate mortar. Then, 1.2 g Na_2_S was mixed and ground to get uniform powder reagents. A 3 mL amount of deionized was mixed with the prepared powdered reagents to get a black slurry, which was calcinated in a muffle furnace at 300 °C at the rate of 10 °C/min for 3 h. The powdered material was filtered, washed, and dried at 105 °C for the fabrication of Ni_3_S_4_/Ag_2_S/TiO_2_/CA aerogel. A similar method was used to synthesize Ag_2_S and Ni_3_S_4_ without TiO_2_.

#### 2.2.3. Synthesis of Ni_3_S_4_/Ag_2_S/TiO_2_/Calcium Alginate Aerogel

Initially, 0.1 g Ni_3_S_4_/Ag_2_S/TiO_2_ was mixed with 4 mL water and stirred for 15 min to make a slurry in a 30 mL glass bottle. After that, 20 mL (2%) of sodium alginate was mixed dropwise with the Ni_3_S_4_/Ag_2_S/TiO_2_ slurry under continuous stirring to make a uniform solution. Then, 6 mL of CaCl_2_ (3%) solution was added to the side wall of the bottle. This causes immediately formation of a gel-like structure, which was left for 3 h to solidify. The obtained Ni_3_S_4_/Ag_2_S/TiO_2_/calcium alginate (Ni_3_S_4_/Ag_2_S/TiO_2_/CA) gel was thoroughly washed with deionized water to remove the excess CaCl_2_. The washed Ni_3_S_4_/Ag_2_S/TiO_2_/Alginate gel was freeze-dried for 36 h at −55 °C in a freeze dryer. The Ni_3_S_4_/Ag_2_S/TiO_2_/CA aerogel was washed with water and ethanol to remove the excessive reagents and dried at 80 °C for 24 h. A similar method was used to synthesize pure CA, Ag_2_S/CA, and Ni_3_S_4_/CA aerogels.

### 2.3. Adsorption Studies

The decontamination of OTC using the prepared materials was investigated in a batch mode by mixing 0.03 g of adsorbent in 30 mL OTC solution at a fixed pH and concentration. The optimum pH identification for OTC removal was studied between pH ranges from 2 to 10. The equilibrium concentration analysis was conducted at 50 mg/L OTC concentration at pH 6 for a 240 min reaction time. The equilibrium time analysis was performed at 5 mg/L and 50 mg/L OTC concentrations at pH 6 between 0 to 240 min. The adsorption capacity (*q_t_*) of the aerogel materials was evaluated using the per unit mass of the adsorbent using the following equation:

(1) $$q_{t}=\frac{(C_{o}-C_{e})V}{W}$$

where *C_o_* and *C_e_* indicate the concentrations of OTC (mg L^−1^) in the solution before and after its removal. The used volume of the OTC is denoted as *V* (L), and the used mass of the aerogel is represented as *W* (g).

### 2.4. Antimicrobial Studies

In this investigation, Ni_3_S_4_/CA, Ag/CA, TiO_2_/CA, and Ni_3_S_4_/Ag_2_S/TiO_2_/CA aerogels were applied for antimicrobial purposes against the native microbial consortium present in the industrial beverage wastewater. Initially, the fresh wastewater sample was filtered through Whatman paper and serially diluted with ultrapure sterile water, inoculated in nutrient agar plates, submerged by a sterile glass spreader, and placed overnight into the incubator at 30 ± 2 °C temperature. After the incubation period on the cultured plates, microbial colonies were observed and counted with the help of a colony counter. These wastewater microbial consortia were saved as test organisms against the synthesized aerogels. These aerogels were directly applied to the diluted wastewater and placed on the magnetic stirrer in sunlight and inside the darkroom. The bacterial count was monitored at different time intervals monitored to measure the effect of the aerogel materials. For this purpose, 100 mL wastewater from each wastewater sample was divided into five 500 mL capacity flasks. An experiment was designed using the following: (i) only food industry wastewater (ii) food industry wastewater and Ni_3_S_4_/CA aerogel (iii) food industry wastewater and TiO_2_/CA aerogel (iv) food industry wastewater and Ag_2_S/CA aerogel and (v) food industry wastewater Ni_3_S_4_/Ag_2_S/TiO_2_/CA aerogel. All flasks were duplicated during the experiment, one series for dark incubation and another for sunlight incubation. The sunlight intensity and atmospheric temperature varied between 650 × 10^2^ to 790 × 10^2^ lux and 35 to 40 °C, respectively. The respective samples were exposed to 0 to 4 h of sunlight or incubated in a dark room simultaneously during the investigation. Subsequently, the turbidity of the solution and bacterial count was measured. In parallel, the antimicrobial potential of synthesized aerogels was analyzed from the microbial consortia obtained from the wastewater sample of food industry sites.

#### 2.4.1. MIC/MBC Values of Aerogels

The antimicrobial potential of synthesized aerogel materials is important because of their potential application and suitability for antiseptic use. The minimum inhibitory concentration (MIC) and minimum bactericidal concentration (MBC) values were determined to investigate the antimicrobial potential of the aerogel material. The MIC is the least amount of aerogel material that prevents the cell formation or the visible growth of bacteria or that acts as bacteriostatic in growth media or solution. The lowest level or concentration of aerogel materials that significantly inhibits the microbial growth is known as the MIC. A minimum dose or concentration of material that causes the death of the microbial population is known as MBC. The MIC and MBC of hybrid aerogels against the indigenous or natural microbial consortia obtained during the microbial count were performed using the broth dilution method previously recommended by the Clinical and Laboratory Standards Institute. The bacterial culture from the food water industries was optimized in Muller Hinton Broth media and set at 0.5 turbidity according to the McFarland standard, indicating a microbial population 1 − 5 × 10^6^ CFU/mL in 0.1 mL solution. The aerogel materials were crushed as a fine powder and suspended at 0, 2, 4, 8, 16, 32, 64,128, 256, and 512 µg/mL in 50 µL of the broth, and with an equal volume of 50 µL of bacterial culture were placed in wells of on a 96 wells plate. The tetracycline antibiotic was used as a positive control in separate wells of the same plate which was then incubated at 35 °C overnight.

#### 2.4.2. Bacterial Zone Inhibition Analysis

Bacterial zone inhibition was conducted using antimicrobial aerogels on the Petri plate solid agar media, also known as the Kirby–Bauer test. This assay is rapid and inexpensive for testing the antimicrobial activity of aerogels. Zone inhibition assay is a qualitative antimicrobial test for antibiotics, chemical compounds, medical fabrics, and other materials used in medical device manufacturing. Previously obtained bacterial culture from the wastewater were purified, and then spread 0.1 mL on the surface of cold agar media plates with the help of a sterile glass rod spreader. The aerogel’s 100 µg were applied to the center of the Petri plate surface. The dish was placed in the incubator for 24 h; during this period, the compound diffuses into the surroundings, preventing bacterial growth around the compound, and creating a zone of inhibition. The size of the zone of inhibition is related to the antimicrobial property of the applied aerogels.

### 2.5. Characterization

The surface morphological studies, elemental analysis, and mapping were carried out through field emission scanning electron microscopy (JEOL, JSM-7600F, FESEM, Tokyo, Japan). The crystallinity and phases of as-prepared samples were analysed by X-ray powder diffractometer (Rigaku, Ultima IV XRD, Tokyo, Japan). The X-ray photoelectron spectroscopy (XPS; ESCALAB 250, Thermo Fisher Scientific, Oxford, UK; used at a monochromatized Al Kα X-ray source λ ¼ 1486.6 eV) was used to detect functional groups and their interactions in the Ni_3_S_4_/Ag_2_S/TiO_2_/CA aerogel. Photoluminescence (RF-5301PC Shimadzu, Kyoto, Japan, spectro-fluorophotometer) and absorbance spectra (UV-visible spectrophotometer; PerkinElmer 750, Waltham, MA, USA) measurements were performed to study the charge recombination and band gap of Ni_3_S_4_/Ag_2_S/TiO_2_/CA aerogel.

## 3. Results and Discussion

### 3.1. Synthesis and Characterization

**Figure 1:**
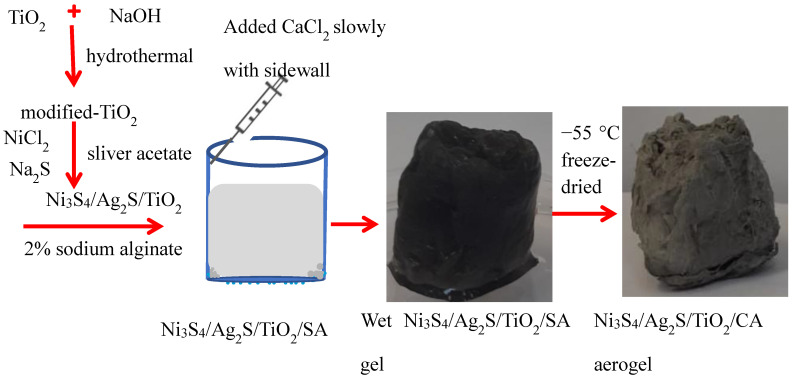
Schematic diagram representing the synthesis of Ni_3_S_4_Ag_2_S/TiO_2_/CA aerogel. The SEM images of Ni_3_S_4_, Ag_2_S and TiO_2_ particles can get an idea of their individual sizes (Figure S1). The SEM images of TiO_2_ show aggregated clusters of nano-ranged particles. Furthermore, the TEM of TiO_2_ shows that nano-ranged particles (<100 nm) are aggregated into clusters of ~500 nm size. The SEM of Ag_2_S shows the majority of particles of sizes in the range of 20–100 nm along with the observance of small clusters of particles with even larger sizes. The SEM of Ni_3_S_4_ shows interconnected globules of micrometer size; however, small clusters of loosely packed particles (approximately in the nanometers range) can be seen protruding out of the globules.

Generally, pure TiO_2_ is a crystalline material with a smooth surface. After treatment with the concentrated NaOH, a mixed phase, like rutile or anatase, and sodium titanate are formed. The mixed phase is generally amorphous with a lamellar structure and defects, providing the surface area to bind with the other semiconductor materials. Moreover, Na^+^ is ion-exchangeable in sodium titanate, which can help in the adsorption or synthesis of new materials using their ion-exchange properties [13,14]. Herein, Ni^2+^ and Ag^+^ ions may replace the Na^+^ ions and form more stable sulfides on the surface of the TiO_2_. A schematic diagram for synthesizing the Ni_3_S_4_/Ag_2_S/TiO_2_/CA aerogel is shown in Figure 1. Several instrumental techniques such as SEM, EDX, elemental mapping, XRD, XPS, etc. have been used to characterize the synthesized materials.

**Figure 2:**
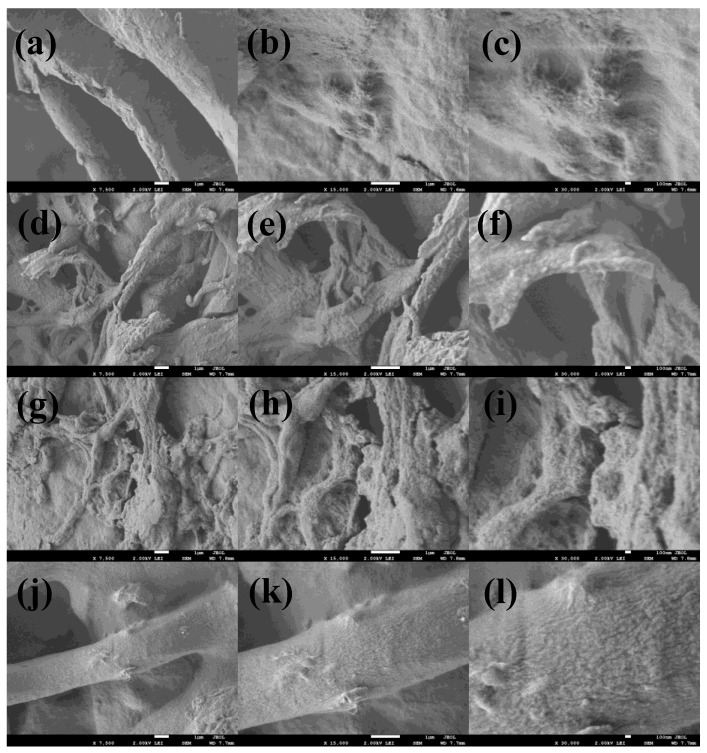
SEM images of CA (**a**–**c**), Ni_3_S_4_/CA (**d**–**f**), Ag_2_S/CA (**g**–**i**) and Ni_3_S_4_/Ag_2_S/TiO_2_/CA (**j**–**l**) at different magnifications.

**Figure 3:**
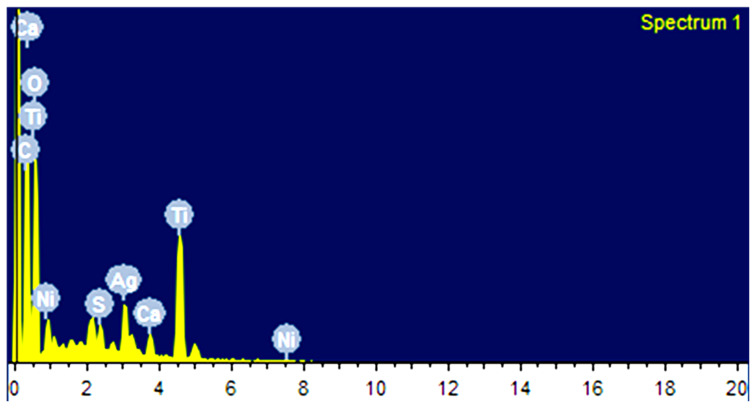
EDAX analysis of Ni_3_S_4_Ag_2_S/TiO_2_/CA.

**Figure 4:**
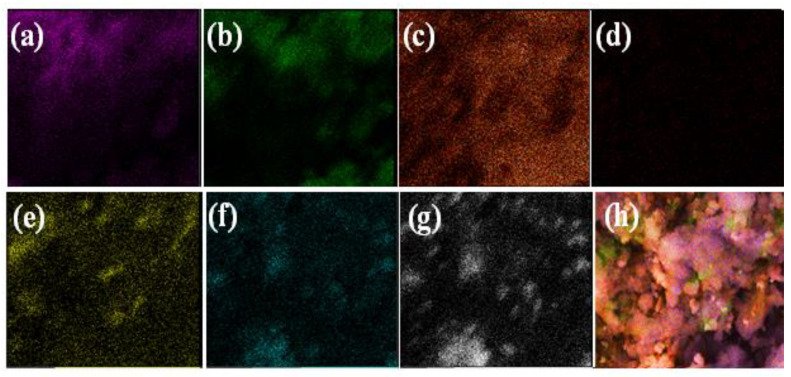
Elemental mapping Ni_3_S_4_/Ag_2_S/TiO_2_/CA. Carbon (**a**), oxygen (**b**), Titanium (**c**), Nickel (**d**), Ca (**e**), Sulfur (**f**), Ag (**g**) and mixed elemental mapping of all elements (**h**).

The morphology of the films was studied by SEM as presented in Figure 2. Pure CA shows a continuous uneven, porous surface with the observance of bulges and small craters. The cross-section seems compact, and the thickness is in the range of 1–1.5 µm. In the case of Ni_3_S_4_/CA (d–f), Ag_2_S/CA (g–i), and Ni_3_S_4_/Ag_2_S/TiO_2_/CA (j–l) the surfaces are relatively much rougher and porous due to the embedded Ni_3_S_4_, Ag_2_S or Ni_3_S_4_/Ag_2_S/TiO_2_. In Ni_3_S_4_/Ag_2_S/TiO_2_/CA, the Ni_3_S_4_/Ag_2_S/TiO_2_ aggregates can be seen protruding out of the CA strands. It can be assumed that Ni_3_S_4_, Ag_2_S, or Ni_3_S_4_/Ag_2_S/TiO_2,_ apart from covering the surface, is also deeply embedded inside the CA network with Ni_3_S_4_, Ag_2_S or Ni_3_S_4_/Ag_2_S/TiO_2_ aggregates protruding out from inside. In contrast to CA, the rougher morphology of Ni_3_S_4_/Ag_2_S/TiO_2_/CA might be due to the nucleation effect of embedded, protruding or surface-covered nanomaterials, which creates depressions or ruptured sites in the CA. The EDAX analysis of Ni_3_S_4_/Ag_2_S/TiO_2_/CA shows the presence of C, O, Ti, Ni, Ag, S and Ca. The non-observance of Na and subsequent presence of Ca suggests successful replacement of Na with Ca during the CaCl_2_ reaction with Ni_3_S_4_/Ag_2_S/TiO_2_/SA (Figure 3). The uniform distribution of C, O, Ti, Ni, Ag, S and Ca in the elemental mapping analysis also suggests the efficacy of the synthesis methodology (Figure 4).

**Figure 5:**
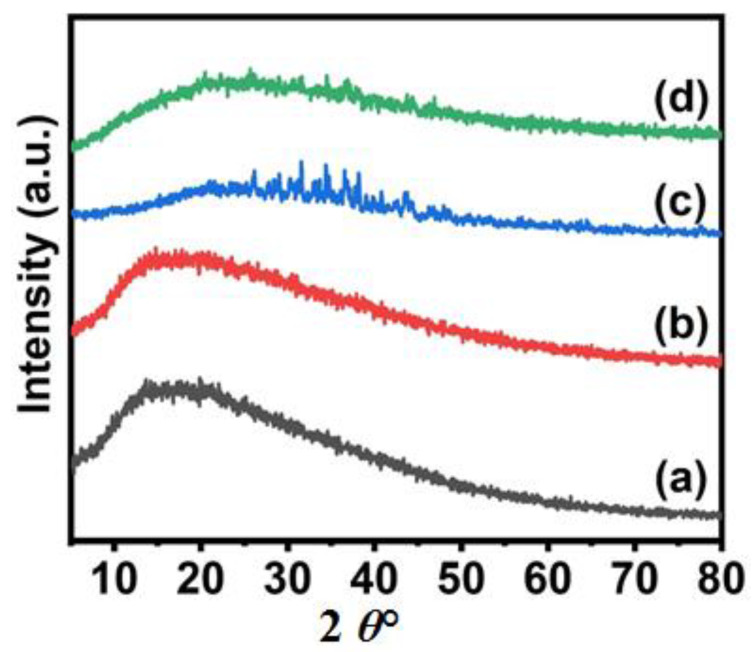
XRD patterns of CA (**a**), Ni_3_S_4_/CA (**b**), Ag_2_S/CA (**c**), and Ni_3_S_4_/Ag_2_S/TiO_2_/CA (**d**).

The XRD pattern of CA, Ni_3_S_4_/CA, Ag_2_S/CA and Ni_3_S_4_/Ag_2_S/TiO_2_/CA are presented in Figure 5. All the samples showed a broad peak in the region of ~20 2θ owing to the presence of amorphous CA and the peaks of Ni_3_S_4_, Ag_2_S, and TiO_2_ are not distinctly observed. Similar reports of highly diffused peaks of fillers in the polymer matrix have been previously reported [14].

**Figure 6:**
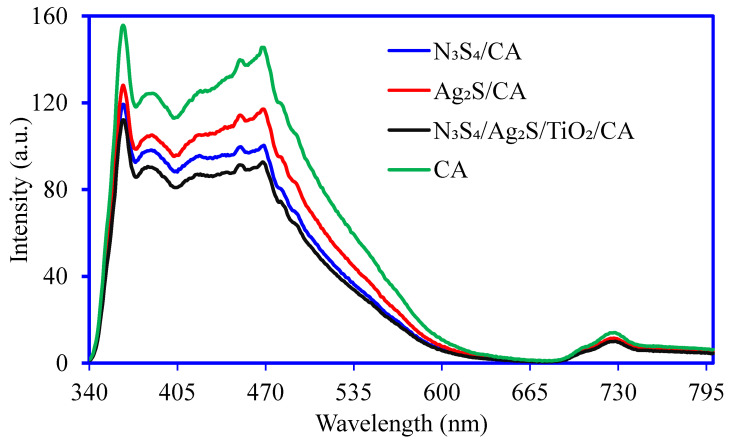
PL emission spectra of CA, Ni_3_S_4_/CA, Ag_2_S/CA, and Ni_3_S_4_/Ag_2_S/TiO_2_/CA.

The PL emission spectra of CA, Ni_3_S_4_/CA, Ag_2_S/CA, and Ni_3_S_4_/Ag_2_S/TiO_2_/CA are presented in Figure 6. The PL spectra are indicative of the charge separation rate and migration efficiency of the charge carriers. The decrease in the PL intensity of Ni_3_S_4_/CA and Ag_2_S/CA compared to CA and a further decrease in loading with Ni_3_S_4_, Ag_2_S and TiO_2_ suggest the highest photocatalytic activity of Ni_3_S_4_/Ag_2_S/TiO_2_/CA. The excitation wavelength of Ni_3_S_4_/Ag_2_S/TiO_2_/CA falls in the visible light region, thereby indicating its visible light activity. The peaks in the region 450–500 nm are related to charge recombination from the conduction band to the recombination center at the ground state. Thus, the lowest intensity peak of Ni_3_S_4_/Ag_2_S/TiO_2_/CA suggests its lowest charge recombination and hence the longer lifetime of the photogenerated carrier [15].

**Figure 7:**
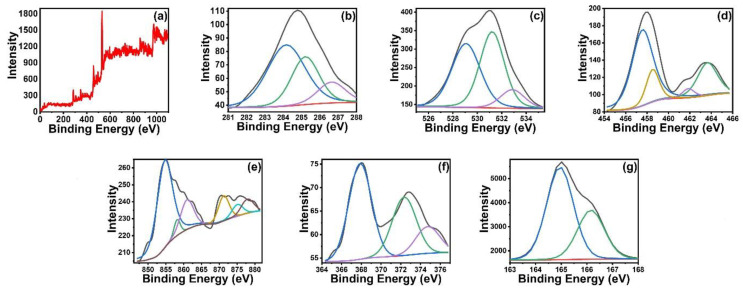
XPS spectra of Ni_3_S_4_/Ag_2_S/TiO_2_/CA. Survey scan (**a**), C1s (**b**), O1s (**c**), Ti2p (**d**), Ni2p (**e**), Ag3d (**f**) and S2p (**g**).

The elemental composition of Ni_3_S_4_/Ag_2_S/TiO_2_/CA studied by XPS as presented in Figure 7 showed the presence of C1s, O1s, Ti2p, Ni2p, Ag3d, Ca2p and S2p peaks corresponding to the presence of oxygen (48.1%), carbon (38.1%), titanium (7.6%), calcium (2.6%), nickel (2.4%), silver (0.9%), and sulfur (0.3%). The C1s spectra can be deconvoluted into 283.80, 284.95 and 288.88 eV peaks corresponding to the C–O–Ti, C=C & C–C, and O–C=O, which correspond to the functionality of the alginate ring and its interaction with Ti and water molecules, respectively [16]. The high percentage of C–C and C–O peaks compared to COOH is due to the major carbon skeleton in rings, C–O in rings, and interconnections between rings and the carbon bonded to the hydroxyl group. The O1s spectra consist of three peaks at 529.07, 531.21 and 532.88 eV corresponding to the oxygen of Ti-O bonds in TiO_2_ (O^2−^ from TiO_2_), C–O and associated water molecules, respectively [17,18,19]. The Ti 2p3/2 and Ti 2p1/2 peaks at 459.42 and 465.68 eV are due to the presence of TiO_2_ [20]. The presence of Ni_3_S_4_ is confirmed by the Ni 2p3/2peak at 854.69, 2p1/2 at 873.4 eV and two shakeup satellite peaks [21,22]. The peaks at 367.88 and 372.34 eV are assigned to Ag 3d5/2 and Ag 3d3/2 of Ag^+^ ions in Ag_2_S, respectively, while the S p1/2 and S p3/2 located at 162.4 and 161.2 eV, respectively, are due to the S of both sulfides [23]. The presence of Ca2p peaks around 360 eV in the survey scan, and subsequently, no observation of Na, confirms the successful replacement of Na by Ca during the composite reaction with CaCl_2_ aqueous solution.

### 3.2. OTC Removal Studies

**Figure 8:**
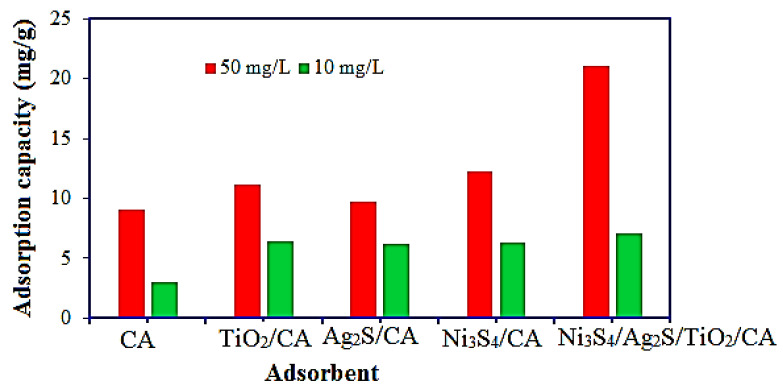
Comparison of the adsorption efficiencies of synthesized aerogels for the removal of OTC. (aerogel mass: 0.03 g, OTC volume: 30 mL, time: 180 min, pH: 6).

The adsorption efficacies of the synthesized aerogels for the removal of the OTC have been evaluated at 10 and 50 mg/L concentrations, and the results are depicted in Figure 8. The OTC adsorption results revealed that the removal capacity of the aerogel increases with the increase in the functionality of the adsorbent. The adsorption capacity of the prepared aerogels is in the following order: Ni_3_S_4_/Ag_2_S/TiO_2_/CA > Ni_3_S_4_/CA > TiO_2_/CA > Ag_2_S/CA > CA. These results revealed that Ni_3_S_4_/Ag_2_S/TiO_2_/CA is the best adsorbent for removing the OTC due to the presence of large functional groups on the surface. Therefore, Ni_3_S_4_/Ag_2_S/TiO_2_/CA aerogel was selected for the brief adsorption studies to identify the optimum experimental condition for OTC removal.

**Figure 9:**
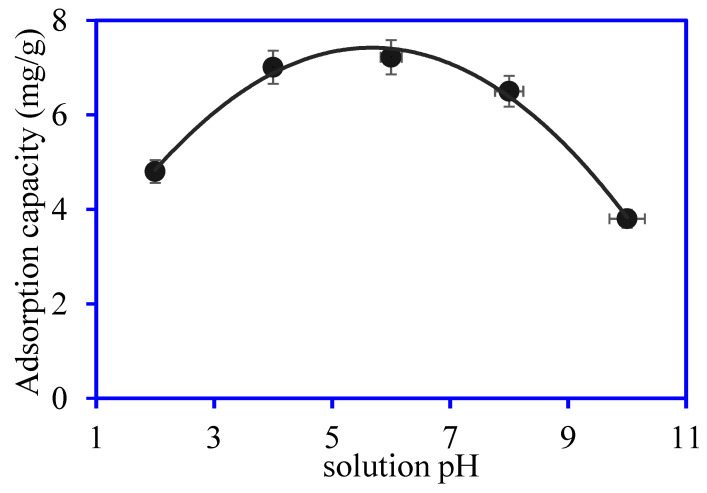
Plot for OTC adsorption at different solution pH onto Ni_3_S_4_/Ag_2_S/TiO_2_/CA aerogel. (OTC conc.: 10 mg/L, aerogel mass: 0.03 g, OTC volume: 30 mL, time: 180 min).

The adsorption of OTC onto Ni_3_S_4_/Ag_2_S/TiO_2_/CA aerogel was investigated at pH ranges from 2 to 10; the results are depicted in Figure 9. The results revealed that solution pH affects the OTC adsorption onto Ni_3_S_4_/Ag_2_S/TiO_2_/CA aerogel, with optimum absorption at pH 6. The optimum adsorption at pH 6 can be explained on the basis of the pKa of OTC. In an acidic medium, OTC is positively charged (OTC^+^) while zwitterionic (OTC^±^) forms in a neutral medium, and the negatively charged molecular form (OTC^−^) is found in a basic medium [24,25]. The pKa values of the OTC ionic forms are 3.57, 7.49, and 9.88. Under the acidic condition (at pH 3), the majority of OTC molecules (80%) exist in the form of cationic (OTC^+^) molecules, which show electrostatic repulsion with the protonated Ni_3_S_4_/Ag_2_S/TiO_2_/CA aerogel. As the solution pH is increased to 6, all the OTC molecules exist in zwitterionic (OTC^±^) form, and the surface charge of the aerogel is close to neutral. The formation of H-bonding and n–π electron donor-acceptor interaction is possible to a more considerable extent due to the presence of the double bond benzene ring, oxygen and nitrogen, sulfur-containing functional groups on OTC molecules and Ni_3_S_4_/Ag_2_S/TiO_2_/CA aerogel [10,26]. Under alkaline conditions, the monovalent and divalent OTC anions become more prominent in the solution, showing an electrostatic repulsion with the deprotonated Ni_3_S_4_/Ag_2_S/TiO_2_/CA aerogel surface, resulting a reduction in the adsorption capacity [27].

**Figure 10:**
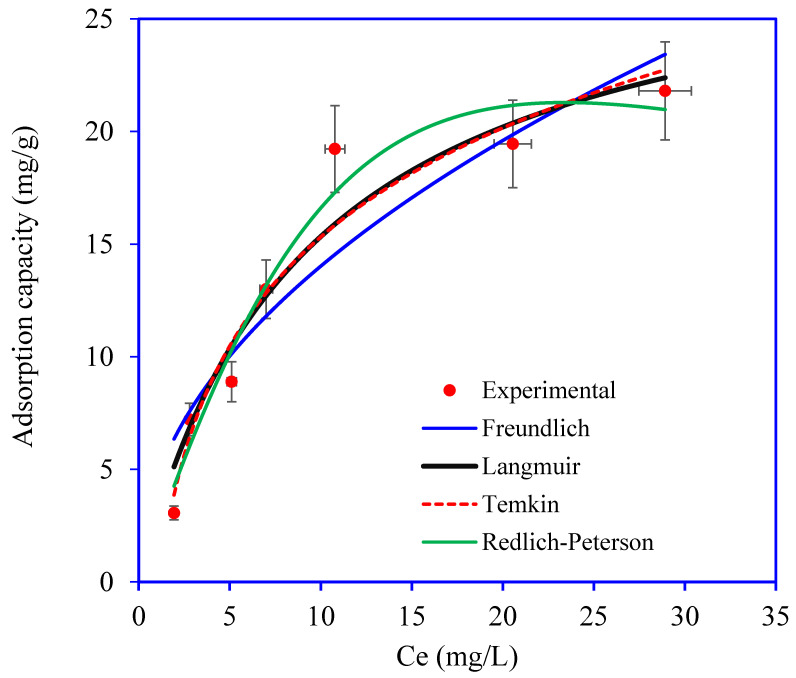
Plot for OTC adsorption isotherms onto Ni_3_S_4_/Ag_2_S/TiO_2_/CA aerogel. (aerogel mass: 0.03 g, OTC volume: 30 mL, time: 180 min, pH: 6).

The adsorption isotherm studies of OTC onto Ni_3_S_4_/Ag_2_S/TiO_2_/CA were investigated at varying initial concentrations between 5 mg/L to 50 mg/L, as shown in Figure 10. The OTC adsorption rate increases with the increase in the OTC molecules in the solution from 5 mg/L to 30 mg/L. A saturation of Ni_3_S_4_/Ag_2_S/TiO_2_/CA active sites has been observed beyond 30 mg/L, meaning that equilibrium between OTC and Ni_3_S_4_/Ag_2_S/TiO_2_/CA has been established. At lower concentrations, OTC molecules are present in fewer numbers, and many active sites on Ni_3_S_4_/Ag_2_S/TiO_2_/CA are available for adsorption. As the ratio between OTC molecules and active sites reduces, Ni_3_S_4_/Ag_2_S/TiO_2_/CA surface sites reach equilibrium [28].

The relationship concerning the Ni_3_S_4_/Ag_2_S/TiO_2_/CA capacity and OTC loading concentration can be discovered by fitting the experimental equilibrium data to Langmuir, Freundlich, Temkin, and Redlich–Peterson isotherm models. The nonlinear equations for the Langmuir, Freundlich, Temkin, and Redlich–Peterson isotherm are as follows:

(2) $$\mathrm{Langmuir}:\;q_{e}=\frac{q_{m}k_{L}C_{e}}{1+k_{L}C_{e}}$$

(3) $$\mathrm{Freundlich}:\;q_{e}=k_{F}C_{e}^{\frac{1}{n}}$$

(4) $$\mathrm{Temkin}:\;q_{e}=B_{t}\;\ln\;(K_{t}C_{e})$$

(5) $$\mathrm{Redlich}–\mathrm{Peterson}:\;q_{e}=\frac{K_{RP}\;C_{e}}{1+\alpha_{RP}\;C_{e}{}^{\beta }}$$

where, *C_e_*: equilibrium concentration, *q_e_*: equilibrium capacity, *q_m_*: monolayer adsorption capacity, *k*_F_ and, 1/*n*: Freundlich constant related to adsorption intensity and adsorption capacity, *K_t_:* equilibrium binding constant, and *B*_t_: the heat of adsorption, $K_{RP}$ (L/g) and $\alpha_{RP}$: Redlich–Peterson isotherm constants and *β*: exponent reflect the heterogeneity of the aerogels. The values of the OTC adsorption isotherm parameters are incorporated in Table 1. The isotherm parameters and error function values are obtained from the plots shown in Figure 10. Based on the lower error functions RMSD, χ^2^ values, and higher R^2^ value, the Redlich–Peterson isotherm model is most suited to the OTC adsorption data onto Ni_3_S_4_/Ag_2_S/TiO_2_/CA. These isotherm modeling results demonstrate that adsorption of OTC occurred on the heterogeneous surface of the Ni_3_S_4_/Ag_2_S/TiO_2_/CA. The Redlich–Peterson isotherm model is the combined model of the Langmuir and Freundlich model. Therefore, OTC adsorption onto Ni_3_S_4_/Ag_2_S/TiO_2_/CA has the characteristics of the Langmuir and Freundlich model.

**Figure 11:**
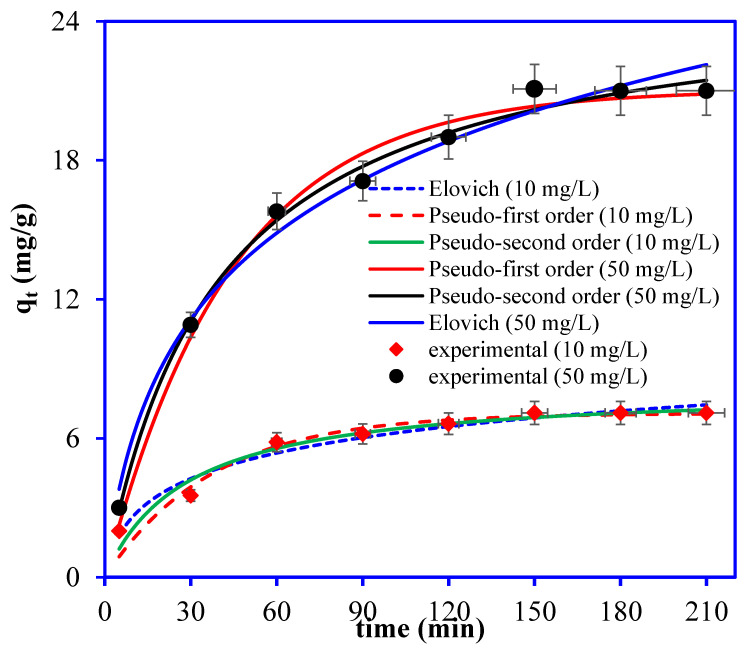
Plot for OTC adsorption kinetics onto Ni_3_S_4_/Ag_2_S/TiO_2_/CA aerogel. (OTC conc.: 50 mg/L, aerogel mass: 0.03 g, OTC volume: 30 mL, time: 180 min, pH: 6).

The OTC adsorption rate onto Ni_3_S_4_/Ag_2_S/TiO_2_/CA as a function of adsorption time was investigated at 10 mg/L and 50 mg/L concentrations. Figure 11 shows the kinetic plot for the OTC adsorption onto Ni_3_S_4_/Ag_2_S/TiO_2_/CA. Initially, OTC was adsorbed rapidly at 10 mg/L concentration, and equilibrium was attained within 60 min due to the large number of vacant sites on the Ni_3_S_4_/Ag_2_S/TiO_2_/CA, while at 50 mg/L concentration, equilibrium was attained within 150 min. Beyond 150 min, adsorption became slower due to the active sites’ saturation [29].

Moreover, to find the rate of OTC removal by Ni_3_S_4_/Ag_2_S/TiO_2_/CA, the kinetic data were applied to the pseudo-first order, pseudo-second order, and Elovich models. The non-linear equations of pseudo-first order, pseudo-second order, and Elovich models are as follows:

(6) $$\mathrm{Pseudo}-\mathrm{first}\;\mathrm{order}:\;q_{t}=qe(1-e^{\left(-k_{1}t\right)})$$

(7) $$\mathrm{Pseudo}-\mathrm{second}\;\mathrm{order}:\;q_{t}=\frac{q_{e}^{2}k_{2}t}{\left[k_{2}\left(q_{e}\right)t+1\right]}$$

(8) $$\mathrm{Elovich}\;\mathrm{models}:\;q_{t}=\frac{1}{\beta }\ln\left(\alpha \beta_{t}\right)$$

where, *q_e_* and *q_t_* are adsorption capacities at equilibrium and at time t (min), *k*_1_ is the pseudo-first order rate constant, *k*_2_ is the pseudo-second order rate constant, *α* and β are adsorption and desorption rate constants for the Elovich model. The plots for the pseudo-first order, pseudo-second order, and Elovich models are included in Figure 11 and the data obtained from the respective plots are depicted in Table 2. Based on higher R^2^ values, lower chi-square (χ^2^), and root-mean-square deviation (RMSD), the Elovich model is the best model for explaining the adsorption of OTC onto Ni_3_S_4_/Ag_2_S/TiO_2_/CA at both concentrations. The Elovich model’s fitting ascribes the OTC adsorption onto Ni_3_S_4_/Ag_2_S/TiO_2_/CA to chemisorption [24,30]. These results reveal that the OTC adsorption rate onto Ni_3_S_4_/Ag_2_S/TiO_2_/CA decreases exponentially with the increase in the OTC amount on the aerogel’s heterogeneous surface [31].

### 3.3. Antimicrobial Studies

**Figure 12:**
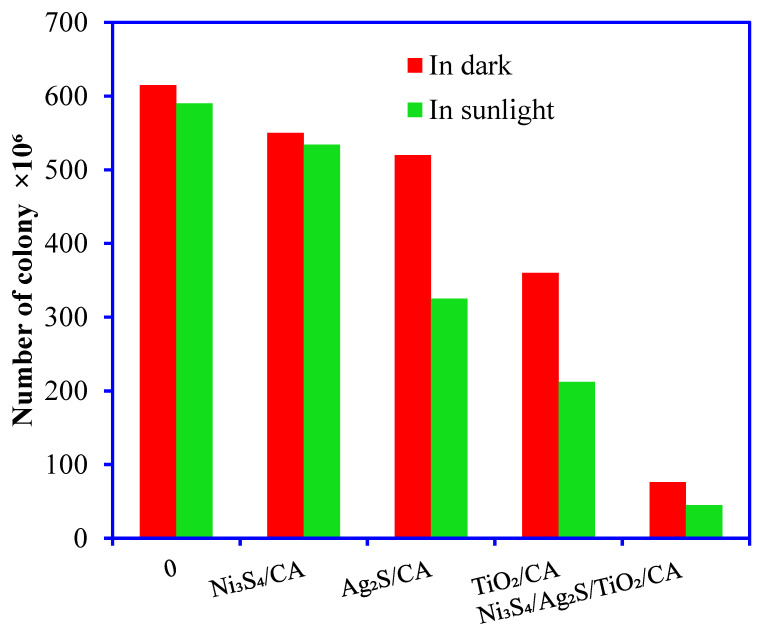
Microbial decontamination properties of Ni_3_S_4_/CA, Ag_2_S/CA, TiO_2_/CA, and Ni_3_S_4_/Ag_2_S/TiO_2_/CA aerogels against the native microbial culture in the absence and presence of sunlight (aerogel dose 100 μg/mL).

**Figure 13:**
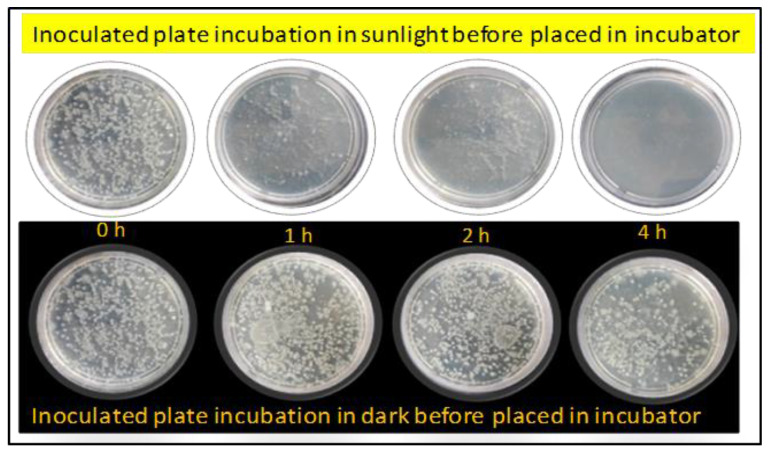
Bacterial growth control properties of Ni_3_S_4_/Ag_2_S/TiO_2_/CA aerogel at different time intervals of 0–4 h in the absence and presence of sunlight (aerogel dose 100 μg/mL).

This study used Ni_3_S_4_/CA, Ag_2_S/CA, TiO_2_/CA, and Ni_3_S_4_/Ag_2_S/TiO_2_/CA aerogels as chemical agents against the bacterial consortium obtained from the industrial beverage wastewater. Initially, the maximum bacterial count was 6.15 × 10^6^ on the plate after the incubation overnight in optimum conditions in the incubator. Further, these microbial consortia were saved as test organisms against the synthesized aerogels. The tested aerogel materials significantly influenced bacterial growth in the absence and presence of sunlight incubation because sunlight activates the aerogels with high electron transportation in the system. After 4 h incubation in sunlight, the test compound led to a maximum decline in colony formation up to 4.5 × 10^5^ in the presence of 100 µg/mL Ni_3_S_4_/Ag_2_S/TiO_2_/CA aerogels in the batch incubation system (Figure 12). Further, the Ni_3_S_4_/Ag_2_S/TiO_2_/CA aerogel was incubated hour-wise with native bacteria in sunlight and dark conditions (Figure 13). Four-hour incubation with aerogel in sunlight led to a complete decline in the bacterial growth. The higher activity of the synthesized aerogels in solar light can be explained on the basis of the photocatalytic activity of the semiconductor materials in the aerogel materials. The semiconductors Ni_3_S_4_, Ag_2_S, TiO_2_ in their respective aerogels can be easily activated by solar light photons, which produce the electron-hole pairs, which react with the surface oxygen and water molecules and produce ^−^O_2_, and OH radicals on the surface of the aerogel. These ^−^O_2_, and OH radicals absorbed by the bacterial cell, break the cell wall, release the nucleic acid, and damage the microbial DNA, resulting in the cell’s death [32].

#### 3.3.1. Bacterial Zone Inhibition

**Figure 14:**
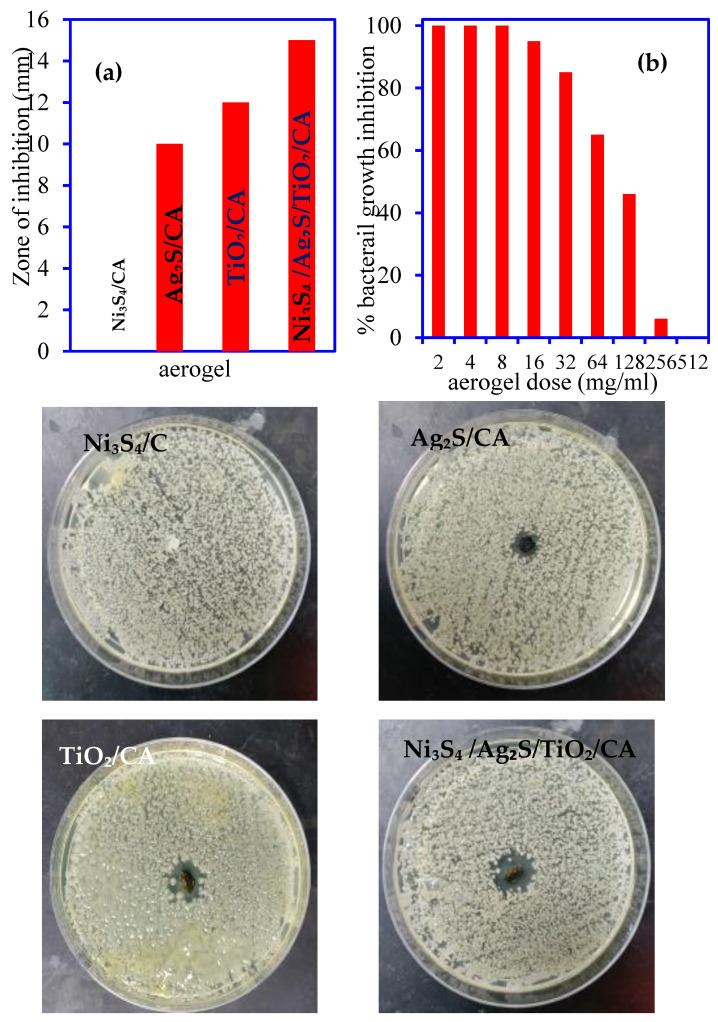
(**a**) Zone inhibition pattern at 100 µg aerogel concentration (zone inhibition in Petri plate) (**b**) effect of Ni_3_S_4_/Ag_2_S/TiO_2_/CA doses on percent bacterial growth inhibition.

Zone inhibition assay reflects the antimicrobial behavior of Ni_3_S_4_/CA, Ag_2_S/CA, TiO_2_/CA, and Ni_3_S_4_/Ag_2_S/TiO_2_/CA aerogels which may be used for medical fabrics and device manufacturing in future. The bacterial culture from the wastewater was spread on the surface of sold agar media plates; 100 µg of each aerogel was applied to the center of a separate Petri plate surface, and left overnight. During this period, the materials diffused and prevented bacterial growth, creating the appearance of a halo or zone. The maximum size of 15, 12 and 10 mm of zone inhibition emerged around the Ni_3_S_4_/Ag_2_S/TiO_2_/CA, TiO_2_/CA, and Ag_2_S/CA materials, respectively, while no zone inhibition occurred around the Ni_3_S_4_/CA (Figure 14a). The higher antimicrobial activity of the Ni_3_S_4_/Ag_2_S/TiO_2_/CA can be explained based of the multiple metals and sulfide in one aerogel which releases more reactive oxygen species (ROS) and kills a higher number of microbial cells [33].

#### 3.3.2. MIC/MBC Analysis

The lowest level or concentration of Ni_3_S_4_/Ag_2_S/TiO_2_/CA aerogel materials significantly inhibiting the microbial growth, known as MIC, was found to be approximately between 64 to 128 µg/mL after overnight incubation at 35 °C. The bacterial growth was halted by more than 50% at 128 µg/mL concentration of Ni_3_S_4_/Ag_2_S/TiO_2_/CA in the growing culture, but sharp growth inhibition was observed from the 64 µg/mL of Ni_3_S_4_/Ag_2_S/TiO_2_/CA (Figure 14b). Hence, the MIC value has occurred between these concentrations and is approx. 96 µg/mL of Ni_3_S_4_/Ag_2_S/TiO_2_/CA aerogel. The microbial decontamination property of the Ni_3_S_4_/Ag_2_S/TiO_2_/CA aerogel increases with the increase of the aerogel dose due to the rise in the active sites in the system. The availability of more active sites releases extra active radicals or species which deactivate the microbe’s cell [34].

#### 3.3.3. Antimicrobial Mechanism

The antimicrobial results revealed that all the synthesized aerogels except Ni_3_S_4_/CA prevent the microbes’ growth and kill them. The effectiveness of the Ni_3_S_4_/Ag_2_S/TiO_2_/CA aerogel was much higher than Ni_3_S_4_/CA, Ag_2_S/CA, and TiO_2_/CA aerogels. The mechanism involved in the microbial deactivation activity of the prepared aerogels may include the destruction of the cell integrity after interaction with the aerogel surface and the release of the reactive oxygen species (ROS). Most current studies assume that the interaction of the microbes with the antimicrobial material may inhibit cell wall/membrane synthesis and interrupt energy transduction [35]. Moreover, antimicrobial material may generate ROS through catalysis, which causes enzyme inhibition and DNA damage. Herein, Ni_3_S_4_/Ag_2_S/TiO_2_/CA aerogel showed microbial decontamination in darkness and solar light irradiation. Under dark conditions, microbes are trapped on the surface of the aerogel, and the interaction between metallic moieties and lipid layers of microbes interrupts the cell respiration and cellular pathways [36]. Moreover, surface interaction between aerogel and microbes inhibits biofilm formation, which prevents microbes’ further growth [35].

**Figure 15:**
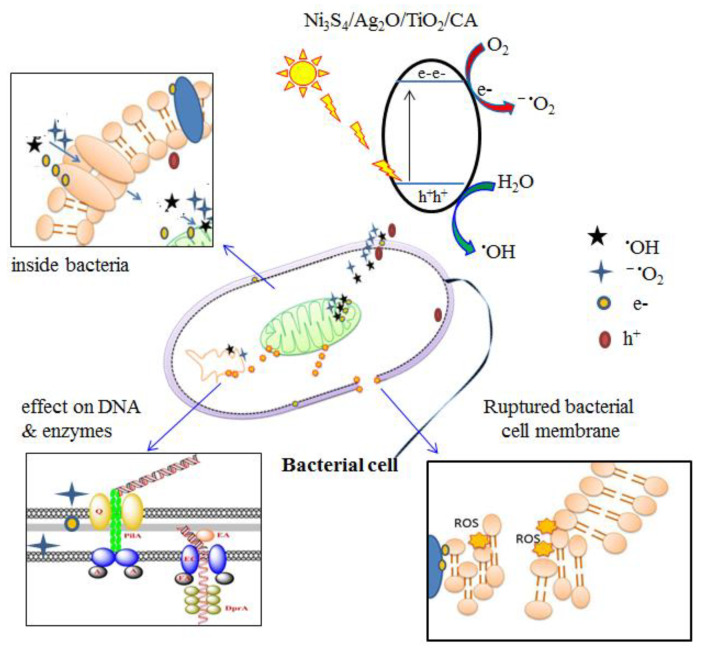
A proposed mechanism for the decontamination of the microbes using Ni_3_S_4_/Ag_2_S/TiO_2_/CA aerogel in dark and solar light.

Under solar light illumination, aerogels work more efficiently due to the production of more active charged and radical species like e^−^, h^+^, ^−^O_2_, OH, and H_2_O_2_. A synergistic effect between Ni_3_S_4_, Ag_2_S, and TiO_2_ in Ni_3_S_4_/Ag_2_S/TiO_2_/CA aerogel has been observed and produced more active radical species, increasing the cytotoxicity within the microbe’s cell and destroying the nucleic acids [37]. The electron transfer mechanism is also expected to occur for the semiconductor Ni_3_S_4_/Ag_2_S/TiO_2_ in Ni_3_S_4_/Ag_2_S/TiO_2_/CA aerogel. The PL analysis (Figure 6) reveals that the PL intensity of the Ni_3_S_4_/Ag_2_S/TiO_2_/CA aerogel is low compared to the other synthesized aerogels, reflecting the better separation of charge carriers, e^−^, and h^+^ [38]. These e^−^ are absorbed by the cell wall and water molecules and generate ROS. This ROS kills the microbial cells [37,39]. A detailed mechanism of microbial decontamination is shown in Figure 15.

## 4. Conclusions

This article reports the successful synthesis of the semiconducting Ni_3_S_4_/CA, Ag_2_S/CA, TiO_2_/CA, and Ni_3_S_4_/Ag_2_S/TiO_2_/CA aerogels for the adsorptive removal of the OTC drug and microbial decontamination of industrial beverage wastewater in the dark and under solar light illumination. The higher adsorption and antimicrobial properties of the Ni_3_S_4_/Ag_2_S/TiO_2_/CA aerogel were mainly due to its surface’s multifunctional groups, which have more active sites for interacting with the pollutants. The maximum adsorption of the OTC was observed at pH 6 and within 180 min of equilibrium time. The maximum monolayer adsorption capacity of the Ni_3_S_4_/Ag_2_S/TiO_2_/CA aerogel was 29.57 mg/g. The adsorption isotherm and kinetic modeling demonstrated that adsorption of the OCT was chemisorption onto the heterogenous surface of the Ni_3_S_4_/Ag_2_S/TiO_2_/CA aerogel. The antimicrobial results of Ni_3_S_4_/CA, Ag_2_S/CA, TiO_2_/CA, and Ni_3_S_4_/Ag_2_S/TiO_2_/CA aerogels indicated that all the materials are active in the dark as well as in solar light except the Ni_3_S_4_/CA aerogel. The zone of inhibition was 10, 12, and 15 mm for Ni_3_S_4_/Ag_2_S/TiO_2_/CA, Ag_2_S/CA, and TiO_2_/CA, respectively. A synergistic effect between Ni_3_S_4_, Ag_2_S, and TiO_2_ in Ni_3_S_4_/Ag_2_S/TiO_2_/CA aerogel has been observed and produced more active radical species, increasing the cytotoxicity within the microbe’s cell and destroying the nucleic acids under the solar light illumination. The photogenerated active charged and radical species such as e^−^, h^+^, −O_2_, OH, and H_2_O_2_ were responsible for the cells’ microbial growth inhibition and killing. The present study demonstrated that Ni_3_S_4_/Ag_2_S/TiO_2_/CA aerogel is the most efficient of these materials for decontaminating the drug and microbes from the wastewater.

## Figures and Tables

**Table 1:** The values of adsorption kinetics parameters for OTC onto Ni_3_S_4_/Ag_2_S/TiO_2_/CA aerogel.

Kinetic Model	Parameters	10 mg/L	50 mg/L
Pseudo-first order:	q_e_ (exp) (mg g^−1^):	7.1	21
	q_e_ (cal) (mg g^−1^):	7.074	21.043
	*k*_1_ (min^−1^):	0.0266	0.022
	R^2^:	0.939	0.983
	RMSE:	0.437	0.651
	χ^2^	1.469	0.412
Pseudo-second order:	q_e_ (cal) (mg g^−1^):	8.216	25.454
	*k*_2_ (g mg^−1^ min^−1^):	0.0042	0.0010
	R^2^:	0.934	0.991
	RMSE:	0.385	0.454
	χ^2^	0.629	0.091
Elovich model:	*a* (mg g^−1^ min^−1^):	0.635	1.054
	*β* (mg g^−1^):	0.587	0.163
	R^2^:	0.944	0.993
	RMSE:	0.353	0.691
	χ^2^	0.219	0.339

**Table 2:** The values of adsorption isotherm parameters for OTC onto Ni_3_S_4_/Ag_2_S/TiO_2_/CA aerogel.

Isotherm Model	Parameters	Values
Langmuir	q_m_ (mg g^−1^):	29.571
	K_L_ (L mg^−1^):	0.107
	R^2^:	0.9370
	RMSE:	1.660
	χ^2^:	1.852
Freundlich	n:	2.069
	K_f_ (mg g^−1^) (mg L^−1^)^−1/n^_F_:	4.609
	R^2^:	0.8739
	RMSE:	2.349
	χ^2^:	3.622
Temkin	B_t_ (J mg^−1^)	553.70
	K_t_ (L mg^−1^):	0.895
	R^2^:	0.9431
	RMSE:	1.577
	χ^2^:	1.354
Redlich–Peterson	$K_{RP}$ (L/g):	2.242
	$\alpha_{RP}$ × 10^−3^ (L/mg):	7.309
	*β*:	1.681
	R^2^:	0.9602
	RMSE:	1.319
	χ^2^:	1.154

## Data Availability

Not applicable.

## Supplementary Materials

The following supporting information can be downloaded at: https://www.mdpi.com/article/10.3390/nano12203642/s1, Figure S1: SEM image of modified TiO_2_ (**a**), Ni₃S₄ (**b**) and Ag_2_S (**c**). TEM image of modified TiO_2_ (**d**).
