# Supplementary material for: Biopolymeric Ni3S4/Ag2S/TiO2/Calcium Alginate Aerogel for the Decontamination of Pharmaceutical Drug and Microbial Pollutants from Wastewater
Source: Nanomaterials (Basel). 2022 Oct 17;12(20):3642. doi: 10.3390/nano12203642 (PMC9609712; doi:10.3390/nano12203642)
Supplement: Supplementary file 1 [file nanomaterials-12-03642-s001.zip › nanomaterials-1940697-supplementary.pdf]

nanomaterials

MDPI

Biopolymeric Ni_3S_4/Ag_2S/TiO_2/calcium alginate aerogel for the decontamination of pharmaceutical drug and microbial pollutants from wastewater

Rajeev Kumar [a],* Mohammad Oves[b], Mohammad Omaish Ansari[c], Md Abu Taleb,[a] M. A. Barakat[a]*,

M.A. Alghamdi[a] and N. H. Al-Makishah[a]

[a]Department of Environmental Sciences, Faculty of Meteorology, Environment and Arid Land Agriculture, King Abdulaziz University, Jeddah-21589, Saudi Arabia
[b]Central of Excellence in Environmental Studies, King Abdulaziz University, Jeddah, Saudi Arabia
[c]Central of Nanotechnology, King Abdulaziz University, Jeddah, Saudi Arabia
*Corresponding Author; R. Kumar; Email: rsingh@kau.edu.sa
M. A. Barakat; Email: mabarakat@gamil.com

Morphological Analysis

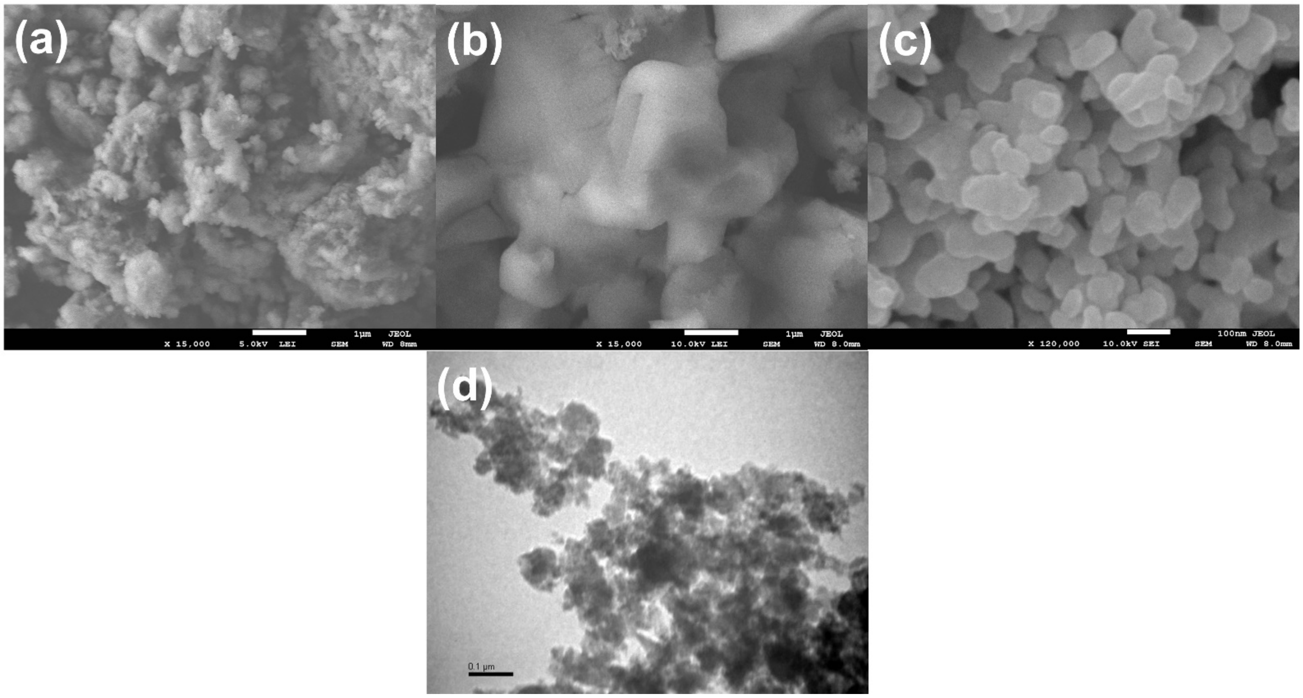

Figure S1. SEM image of modified TiO_2 (a), Ni_3S_4 (b) and Ag_2S (c). TEM image of modified TiO_2 (d).
